# The relationship between the lifestyle health index and voter turnout during the 2020 United States presidential election in the context of regional cultures

**DOI:** 10.1016/j.puhip.2024.100534

**Published:** 2024-08-15

**Authors:** Ross Arena, Nicolaas P. Pronk, Thomas E. Kottke, Anthony Arena, Colin Woodard

**Affiliations:** a Department of Physical Therapy, College of Applied Science, University of Illinois, Chicago, IL, USA; b Healthy Living for Pandemic Event Protection (HL – PIVOT) Network, Chicago, IL, USA; c HealthPartners Institute, Minneapolis, MN, USA; d Department of Health Policy and Management, University of Minnesota, Minneapolis, MN, USA; e Walter Payton High School, Chicago, IL, USA; f Nationhood Lab, Pell Center for International Relations and Public Policy, Salve Regina University, Newport, RI, USA

**Keywords:** Unhealthy lifestyle behaviors, Chronic disease, Social vulnerability, Public health

## Abstract

**Objectives:**

There are numerous population health challenges confronting the United States (U.S.), including the unhealthy lifestyle – chronic disease pandemics. However, the impact of unhealthy lifestyle behaviors and the increased prevalence of chronic diseases that result from them affect many facets of life outside of the health domain, and their scope remains under-appreciated. The current analysis contributes to addressing this knowledge gap by comparing the newly developed Lifestyle Health Index (LHI) to U.S. county-level voter turnout rates in the 2020 presidential election.

**Study design:**

Descriptive, cross-sectional, retrospective analysis.

**Methods:**

County-level data on the LHI, percent voter turnout, and the American Nations regional cultures model schematic was used in the current analysis.

**Results:**

Pearson correlations between county-level LHI scores and sub scores and Democratic, Republican, and overall voter turnout were all statistically significant and of similar strength (*r* > 0.63, *p* < 0.001). All counties in the worst performing LHI quartile had a voter turnout <60 %. Higher LHIs were consistently assocaited with lower voter turnout across the regional cultures, although heterogeneity was evident across the American Nations.

**Conclusions:**

A large percentage of the U.S. population is afflicted with poor health, and unhealthy lifestyle behaviors are a primary driver. Poor health does not occur in a vacuum and impacts many other facets of an individual's life. The current study further demonstrates the potential detrimental impact of poor health on civic engagement, specifically participation in the electoral process (i.e, citizens' health may influence voter turnout). Health care professionals and institutions in the U.S. should uniformly embrace the recent policy brief by the American College of Physicians on participation in the electoral process for patients receiving care. This paradigm shift has the potential to substantially improve voter turnout during U.S. elections.

## 1 Introduction

The unhealthy lifestyle and chronic disease pandemics are currently two of the leading population-level health challenges facing the United States (U.S.), and there is no viable solution on the horizon for either [[1], [2], [3]]. In fact, the unhealthy lifestyle – chronic disease crises are core components of a leading syndemic [4,5], meaning these issues are intricately linked and must be addressed simultaneously. The societal impacts of these individual pandemics and broader syndemic extend beyond health outcomes and burdens on the health care system as has been previously shown for workplaces and other economic entities [6,7].

A healthy democratic society requires robust citizen participation in voting and other civic processes. Unfortunately, voter turnout in the U.S. lags that of many peer democracies. The Pew Research Center reported that approximately 66 % of eligible voters participated in the 2020 Presidential Election [8]. While this was a historically high participation rate in a U.S. national election, it ranked 31st of 49 national elections held recently around the world [9]. A half century of political science research has shown voter turnout is associated with income, age, and especially educational attainment [[10], [11], [12]].

There is evidence to indicate health also impacts U.S. voter turnout, although additional study is needed to better understand the relationship. The current analysis furthers this work by comparing the newly developed Lifestyle Health Index (LHI) [13] to U.S. county-level voter turnout rates for the 2020 presidential election. The analysis was performed at the national level, independently by Democrat vs. Republican voter majority counties, and distinct geographical regions with differing cultural values according to the American Nations model [14]. A primary goal of the current analysis was to further explore the relationship between citizens’ health and voter turnout.

## 2 Methods

### 2.1 Study Design

Descriptive, cross-sectional, retrospective analysis.

### 2.2 Lifestyle health index data source and calculation

We obtained 2021 county-level, age-adjusted risk behaviors, health outcomes and health status prevalence data from the 2023 Centers for Disease Control and Prevention (CDC) PLACES database [15]. These PLACES data were generated by the Behavioral Risk Factor Surveillance System survey [16]. The method of calculation for the *Lifestyle Health Index (LHI)* is as follows:

*Sum of county level prevalence for:* [***Risk Behaviors*** *(Binge drinking + Current smoking + No leisure-time physical activity + Sleeping less than 7 h)]* + *[****Health Outcomes*** *(High blood pressure + Cancer (excluding skin cancer) + Coronary heart disease + Chronic obstructive pulmonary disease + Diagnosed diabetes + Chronic kidney disease + Obesity among + Stroke among)]* + *[****Health Status*** *(Fair or poor self-rated health status + Mental health not good for≥14 days + Physical health not good for≥14 days)].* [13].

The overall LHI score as well as Risk Behavior, Health Outcome and Health Status sub scores were all calculated for analysis in the current study. For the LHI overall and sub scores: 1) Percentage prevalence is in decimal form (i.e., X/100) for each measure in sum score; 2) All prevalence values are age-adjusted for adults ≥18 years of age. Hence, LHI scores may range between 0 (ideal health – 0 % prevalence for all LHI measures) and 15 (poorest health – 100 % prevalence for all LHI measures).

### 2.3 Social vulnerability

The 2020 CDC/Agency for Toxic Substances and Disease Registry (ATSDR) Social Vulnerability Index (SVI) database provides overall and subtheme SVI scores [17]. It seeks to capture the potential negative effects on communities due to external health stressors using 16 U.S. census variables. Data used to calculate the 2020 CDC/ATSDR SVI was derived from 5-year (2016–2020) American Community Survey data [18].

### 2.4 Percent voter turnout

Data on percentage of the U.S. adult population (i.e., ≥18 years) who voted in the 2020 presidential election were obtained from the 2023 County Health Rankings (CHR) program of the University of Wisconsin Population Health Institute [19]. Source data for county-level percent voter turnout came from the MIT Election Data and Science Lab. [20]. County-level results for the 2020 U.S. presidential election in the context of Republican vs. Democrat candidate vote total were obtained directly from the MIT Election Data and Science Lab. [20].

### 2.5 Regional cultures

**Fig. 1:**
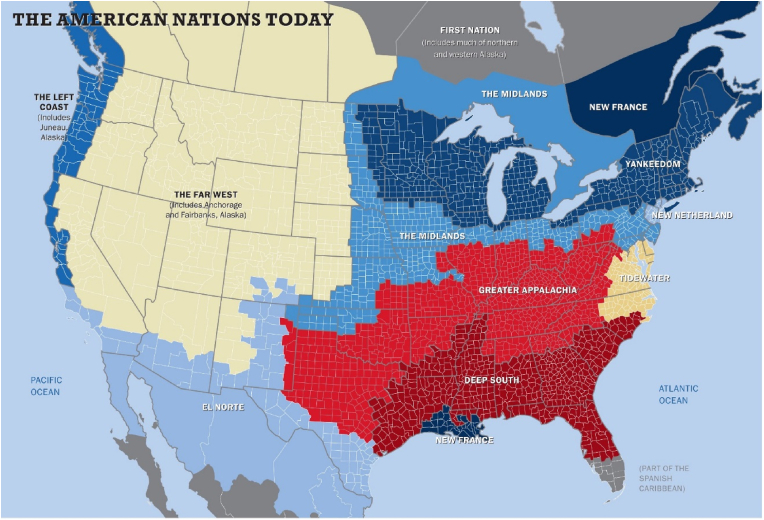
The American Nations Model. Reprinted with permission: https://www.nationhoodlab.org/a-balkanized-federation/.

The *American Nations* regional model was obtained from the Nationhood Lab [21]. Cultural geographers have long recognized First Settler effects on the characteristics of national and regional cultures, with Wilbur Zelinsky's “Doctrine of First Effective Settlement” [22] arguing that “the dominant culture of a given nation is determined by the characteristics of the first group of settlers … regardless of how small the initial band of settlers might have been.” Regional cultures can thus be discerned and mapped by tracking competing first settlement streams, an exercise that has informed the work of historians and geographers [[23], [24], [25]]. The American Nations model [14] uses this approach to more accurately designate U.S. regions and has been applied to explain differences in several areas including economic development [26], mortality [27], gender wage gaps [28], personality characteristics [29], voting behavior [30], social vulnerability [31], and health characteristics [32]. A detailed description of the *American Nations* cultures has been published [33]. The American Nations cultural model is illustrated in Fig. 1, and descriptions of the unique cultural characteristics of the American Nations have been previously published [32].

### 2.6 Process for merging databases

The CHR, CDC PLACES, MIT, and Nationhood Lab databases were linked through zip-code identifiers using Microsoft Excel (Redmond, WA).

### 2.7 Subject protection

HealthPartners Institute Research Subjects Protection Program determined that this study is exempt from IRB review and ongoing oversight under 45 CFR Part 46 as it involves the analysis of existing, publicly available data.

### 2.8 Statistical analysis

Pearson Product Moment correlation coefficients assessed the relationships between the LHI overall score and sub scores and county-level voter turnout. Correlations were assessed in all U.S. counties as well as subgroups according to Democratic and Republican County majorities. Correlations between the overall SVI and both the overall LHI and county-level percent voter turnout were also determined. Independent t-tests were used to compare: (1) LHI overall and sub scores; and (2) County-level percent voter turnout according to Democratic and Republican candidate majority grouping on a county level. Analysis of variance (ANOVA) with Fisher's least significant difference post-hoc testing was used to assess county-level percent voter turnout according to overall LHI quartiles. ANOVA comparisons by LHI quartiles were performed for all U.S. counties as well as subgroups according to Democratic and Republican majority counties. County-level LHI scores and percent voter turnout were morphed into the respective cultural regions and reported according to the American Nations model to identify areas with particularly high LHI scores in conjunction with low percent voter turnout. All statistical tests with a *p*-value <0.05 were considered significant. SPSS (Version 28, IBM, Armonk, NY) was used for all statistical analyses.

## 3 Results

Data were available from 3062 U.S. counties - Florida did not report the data needed to calculate the LHI in 2021 and was therefore excluded from the current analysis.

**Fig. 2:**
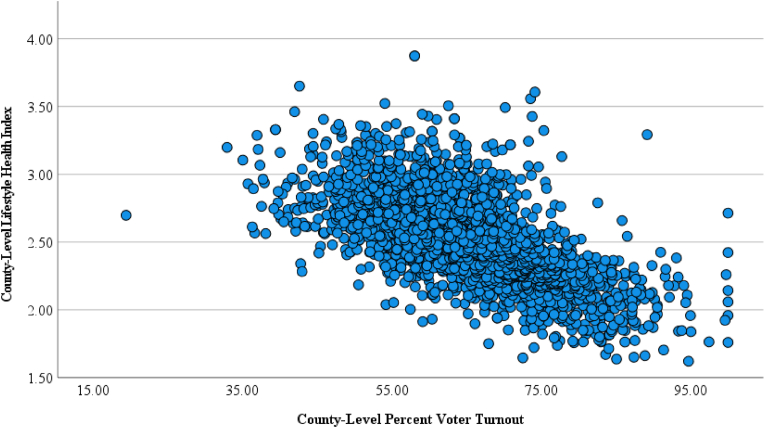
County Level Lifestyle Health Index – Percent Voter Turnout Scatterplot. **Legend**: *r* = −0.686, <0.0001.

Table 1 lists the correlations between LHI overall and sub scores and voter turnout in the entire cohort as well as subgroups based on Democrat vs. Republican vote majority on a county level. All correlations were statistically significant, and the relationships were consistent across all analyses. Fig. 2 illustrates the scatter plot of the correlation between overall LHI and percent voter turnout in the entire cohort. The correlations between overall SVI and: 1) LHI (*r* = −0.63, *p* < 0.001); and 2) county-level voter turnout (*r* = −0.58, *p* < 0.001) were statistically significant.

**Table 1:** Pearson product moment correlation: Lifestyle health index and county-level percent voter turnout in the 2020 U.S. Presidential election.

		County-Level Percent Voter Turnout: Overall Group (*n* = 3062)	County-Level Percent Voter Turnout: Democratic Candidate Majority (*n* = 535)	County-Level Percent Voter Turnout: Republican Candidate Majority (*n* = 2527)
**LHI – Risk Behavior Subscore**	Pearson Correlation	−0.638	−0.646	−0.641
	*p*-value	<0.0001	<0.0001	<0.0001
**LHI – Health Outcomes Subscore**	Pearson Correlation	−0.641	−0.626	−0.671
	*p*-value	<0.0001	<0.0001	<0.0001
**LHI – Health Status Subscore**	Pearson Correlation	−0.648	−0.649	−0.648
	*p*-value	<0.0001	<0.0001	<0.0001
**LHI – Overall Score**	Pearson Correlation	−0.668	−0.652	−0.686
	*p*-value	<0.0001	<0.0001	<0.0001

**Legend:** LHI, lifestyle health index.

Table 2 lists t-tests results comparing LHI overall and sub scores and percent voter turnout according to Democratic vs. Republican vote majority counties. All comparisons were significantly different with poorer LHI scores and lower voter turnout in the Republican vote majority subgroup.

**Table 2:** Independent T-Test results comparing voter turnout and the LHI.

		Democratic Candidate Majority (*n* = 535)	Republican Candidate Majority (*n* = 2527)
**LHI – Risk Behavior Subscore**	Mean (SD)	0.93 ± 0.15*	0.99 ± 0.09
**LHI – Health Outcomes Subscore**	Mean (SD)	1.03 ± 0.21*	1.07 ± 0.11
**LHI – Health Status Subscore**	Mean (SD)	0.45 ± 0.11*	0.47 ± 0.08
**LHI – Overall Score**	Mean (SD)	2.42 ± 0.46*	2.53 ± 0.27
**County-Level Percent Voter Turnout**	Mean (SD)	67.83 ± 10.93*	65.14 ± 9.78

**Legend:** LHI, lifestyle health index; SD, Standard Deviation.

All differences significantly different, *p* < 0.001.

Table 3 lists ANOVA results comparing percent voter turnout according to LHI quartiles in the overall group as well as Democrat and Republican vote majority subgroups. The differences were significant for all comparisons in the overall group and subgroups – a progressively higher (i.e., poorer) LHI was associated with a progressively lower percent voter turnout.

**Table 3:** Analysis of variance comparison of voter turnout according to LHI quartiles.

	LHI Quartile	N	Mean*	Std. Deviation	Std. Error
**County-Level Percent Voter Turnout: Overall Group**	<2.29	744	75.38	7.74	0.28
	2.29–2.47	783	68.27	6.91	0.25
	2.48–2.71	771	61.50	7.40	0.27
	≥2.72	764	57.51	7.67	0.28
	**Total**	**3062**	**65.61**	**10.04**	**0.18**
**County-Level Percent Voter Turnout: Democratic Candidate Majority**	<2.08	130	78.55	7.86	0.69
	2.08–2.28	138	71.14	7.12	0.61
	2.29–2.74	133	63.07	7.42	0.64
	≥2.75	134	58.75	9.13	0.79
	**Total**	**535**	**67.83**	**10.93**	**0.47**
**County-Level Percent Voter Turnout: Republican Candidate Majority**	<2.32	581	74.95	7.48	0.31
	2.32–2.50	704	67.61	6.84	0.26
	2.51–2.71	613	61.11	7.38	0.30
	≥2.72	629	57.24	7.30	0.29
	**Total**	**2527**	**65.14**	**9.78**	**0.19**

**Legend:** LHI, lifestyle health index.

*All percent voter turnout differences statistically significant for overall and subgroup LHI Quartiles – *p* < 0.001.

Table 4 lists LHI overall and sub scores and percent voter turnout according to the American Nations model. Key observations from Table 4 are: 1) Heterogeneity in LHI and percent voter turnout was apparent across the American Nations; and 2) Higher LHI overall and sub scores were consistently associated with low percent voter turnout. The Deep South, New France, El Norte, and Greater Appalachia had the highest LHI and lowest percent voter turnout.

**Table 4:** Average lifestyle health index subscores and overall score in the American nations.

	Number of Counties	LHI – Risk Behavior Subscore	LHI – Health Outcomes Subscore	LHI – Health Status Subscore	LHI – Overall Score	Regional Geographic-Level Percent Voter Turnout
**Deep South**	452	1.06	1.21	0.54	2.82	60.37
**El Norte**	89	0.97	1.07	0.53	2.57	59.98
**Far West**	430	0.90	0.96	0.43	2.28	71.57
**Federal Entity**	1	0.80	0.83	0.35	1.97	65.22
**Greater Appalachia**	939	1.02	1.11	0.51	2.64	61.19
**Greater Polynesia**	4	0.90	0.78	0.36	2.05	59.89
**Left Coast**	54	0.81	0.89	0.42	2.12	75.77
**Midlands**	469	0.97	1.03	0.42	2.42	66.77
**New France**	27	1.08	1.16	0.53	2.78	63.50
**New Netherlands**	25	0.86	0.85	0.37	2.08	70.98
**Tidewater**	131	0.93	1.07	0.44	2.45	70.55
**Yankeedom**	441	0.93	0.97	0.40	2.30	71.64
**Total**	**3062**	**0.98**	**1.06**	**0.47**	**2.51**	**65.61**

**Legend:** LHI, lifestyle health index.

*Estimates for Florida are not available for measures based on BRFSS 2021. Florida was unable to collect data over enough months to meet the minimum requirements for inclusion in the 2021 annual aggregate data set (https://www.cdc.gov/places/help/data-notes/).

## 4 Discussion

The current investigation substantially adds to the existing literature linking health characteristics to voter turnout in the U.S. through a county-level analysis of the 2020 Presidential election. Primary findings from the analysis herein are as follows: 1) There is a strong association between percent voter turnout and the newly proposed LHI, suggesting poor health may be a powerful contributor to lower participation in the voting process. This was consistent for both the overall LHI and sub scores, the latter of which individually reflects the prevalence of unhealthy lifestyle behaviors, perceived health status and chronic disease diagnoses. The consistent strength of the correlation between all LHI calculations and percent voter turnout indicate a host of health metrics are potentially important drivers of participation in the voting process. LHI scores in the highest quartile demonstrate less the 60 % voter turnout. Moreover, the relationship between LHI and county-level voter turnout was stronger than the relationship between overall SVI and county-level percent voter turnout, although both correlations were statistically signficant. This observation indicates both health status and measures of social vulnerability may be important drivers of voter turnout; 2) Counties that had a majority vote for the Republican candidate for the 2020 U.S. Presidential Election had a significantly higher LHI and significantly lower percent voter turnout. However, the strength of correlation between all LHI calculations and percent voter turnout was similar in Republican and Democratic county sub groups. As with the overall group comparisons, voter turnout is <60 % in both Democratic and Republican counties with LHI scores in the highest quartile. These findings indicate the influence of health characteristics on voter participation transcends political ideology; and 3) The American Nations model demonstrates stark differences in LHI and percent voter participation across regions with unique cultural identities. A higher LHI coincides with lower percent voter turnout. Several regions defined by the American Nations are particular areas of concern with poor population health and voter turnout well below the national average for the 2020 U.S. Presidential election. This would indicate that the nature of the health-civic engagement relationship varies by regional culture – at least as defined by the American Nations.

What should be the role of health care professionals and institutions in promoting civic engagement of the patients under their care? A recent policy brief from the American College of Physicians (ACP) [34] puts forth the following recognitions and recommendations: “(1) ACP recognizes that voting impacts health and health care; (2) ACP supports policies that ensure safe and equitable access to voting and opposes the institution of barriers to both the process of voter registration and the act of casting a vote; (3) ACP supports the drawing of fair, representative, and nonpartisan electoral districts. ACP recognizes that partisan gerrymandering may exacerbate health inequities through the disenfranchisement of vulnerable communities and supports efforts to end the practice of partisan gerrymandering; (4) ACP encourages medical students, residents, physicians, and other health care professionals to vote and supports efforts to eliminate barriers to their participation in the electoral process; and (5) ACP encourages nonpartisan health care–sponsored voter engagement as a strategy to increase health equity for patients and health care professionals” [34]. The findings of the current study provide data strongly supportive of these recognitions and recommendations. Because participation in the voting process is lower among those with poor health – a cohort who are more likely to be regularly interacting with health care professionals – ACP's policy brief presents a framework for intervention [34]. The findings in this study suggest that (non-partisan) voter registration and mail-in ballot initiatives could be introduced in health care settings and would have the potential to substantially improve voter participation. The Health Democracy Kit (HDK) [35] is a toolkit specifically designed for healthcare settings. The toolkit contains a badge and posters that contain QR and text codes that direct patients to an online platform where they can register to vote and request mail-in ballots. In one initiative conducted between May and November 2020, over 13,000 health care professionals and students from approximately 2400 health care settings ordered 24,031 individual HDKs [35]. Representatives from approximately 600 health-focussed institutions ordered 960 institutional HDKs. All 50 states and the District of Columbia were represented in this initiative. Collectively, the HDKs assisted in facilitating 27,317 voter registrations and 17,216 mail-in ballot requests. Another initiative, Vot-ER, “develops nonpartisan civic engagement tools and programs for every corner of the healthcare system—from private practitioners to medical schools to hospitals” [36]. Vot-ER provides badges for health care professionals to wear bearing a scannable QR code that links to the non-partisan Vot-ER platform where individuals can register to vote, request a mail-in ballot, and receive information on upcoming elections [37]. Ruxin et al. [38] reported that medical student-led teams (128 from 80 medical schools across 31 states and the District of Columbia) using the Vot-ER platform helped 15,692 adults initiate voter registration and/or mail-in ballot requests. In a post-campaign survey of medical student captains (64.1 % response rate), the primary motivation to participate was to promote social justice. The majority of medical school captain respondents reported they would include voter registration campaigns in their clinical practice moving forward (92.7 %). Junior et al. [39] reported on a Vot-ER initiative in pediatric settings across the U.S. (1490 health professionals and students in 353 pediatric institution), facilitating 389 voter registrations, although the authors posit this was an underestimation of actual registrations through this campaign. Findings from these initiatives support the ability of health care professionals and institutions to improve voter participation through registration and mail-in ballot request campaigns, both of which can be scaled. In 2022, the U.S. Bureau of Labor Statistics estimate 14.7 million individuals 16 years and older were employed in healthcare occupations [40]. There are 6120 hospitals (in 2024) [41] and more that 48,000 outpatient clinics (in 2022) [42] in the U.S. Given the scale of its workforce and infrastructure, this sector has substantial potential to improve voter turnout among their patients.

Pronk et al. [43] recently reported that poor health, as reflected by an index of 12 public health outcomes, was strongly associated with reduced state-level voting access. These findings in conjunction with the current analysis indicate poor health may in and of itself be creating barriers to voting and is compounded by reduced voting access in areas where a higher percentage of the population is in poor health. The effects of poor health may be compounding in multiple directions, i.e., poorer health is associated with lower voring turnout as well as with reduced voting access. This possible synergistic barrier to voting makes the role of health care professionals and institutions, through the adoption of initiatives such as Vot-ER [38] and HDK [35], all the more vital.

Improving the health of the U.S. population is a non-partisan endevour; irrespective of political ideology, all efforts should be made to improve an individuals/communities health trajectory and promote civic engagement. Moreover, efforts should be made to tailor health and civic engagement messaging and programs to resonate within specific communities. Our findings indicate the impact of health status on voter turnout may be similar across the two leading U.S. political parties (i.e., Democrat and Republican), despite the observation that Republican majority counties demonstrated a higher LHI and lower percent voter turnout compared to Democrat majority counties. Further study is needed to understand the causes and solutions for health and civic engagement disparities between Republican and Democratic majority counties. Thoughtfully tailored messaging and initiatives that consider a given community's value, belief, and ideological systems are more likely to be successful.

Previous publications have demonstrated the heterogenicity in health status across the U.S. using the American Nations model [31,32,44]. This model proposes that several distinct regional cultural identities exist within the U.S., and these cultural identities have been shown to have an enormous influence over a wide range of phenomena, including population health. Our findings presented in Table 4 indicate several American Nation regions are of particular concern with respect to both population health and civic engagement. Health-related messaging and communications may need to be considered according to the values, norms, and beliefs of the respective regional culture in order to be successful at engaging the population.

It is important to note that the current analysis demonstrates signficant relationships (i.e., correlations) between LHI and voter turnout. While these relationships allow us to posit that the LHI may *predict* voter turnout, reverse causality cannot be completely discounted (i.e., longitudinal voting behaviors may influence an individuals health patterns). Clearly, additional research is needed in this area to better understand the relationship between citizens’ health and voting behaviors and determine the direction(s) of causality.

In conclusion, a large percentage of the U.S. population is afflicted with poor health. Unhealthy lifestyle behaviors are a primary driver of the chronic disease pandemic/syndemic we face. The newly proposed LHI comprehensively illustrates the magnitude of this crisis. The current study further demonstrates the detrimental impact of poor health on civic engagement and notes an important linkage to regional cultures that underscores the complexity of the health-civic engagement relationship. Health care professionals and institutions in the U.S. should consider embracing the recommendations in the recent policy brief [34] by the ACP on participation in the electoral process for patients receiving care. This paradigm shift has the potential to substantially improve voter turnout during U.S. elections on all levels of government.

## Ethical approval

HealthPartners Institute Research Subjects Protection Program determined that this study is exempt from IRB review and ongoing oversight under 45 CFR Part 46 as it involves the analysis of existing, publicly available data.

## Funding

None.

## Author contributions

All authors had access to the data. RA prepared the initial draft of the manuscript. RA and CW prepared data analysis. TK, NP, and AA provided critical revisions and added content to the manuscript draft.

## Declaration of competing interest

The authors declare that they have no known competing financial interests or personal relationships that could have appeared to influence the work reported in this paper.
